# Domestic *Triatoma* spp. Infections with *Trypanosoma cruzi*, Household Infestations, and Molecular Identification in Oaxaca, México

**DOI:** 10.3390/insects13121134

**Published:** 2022-12-08

**Authors:** Nadia A. Fernández-Santos, Josefina C. Trujillo-García, Sarah A. Hamer, Lihua Wei, Humberto Martínez-Montoya, Patricia Tamez-Guerra, Gabriel L. Hamer, Mario A. Rodríguez-Pérez

**Affiliations:** 1 Instituto Politécnico Nacional, Centro de Biotecnología Genómica, Reynosa 88170, Mexico; 2 Department of Entomology, Texas A&M University, College Station, TX 77843, USA; 3 Secretaría de Salud, Laboratorio Estatal de Salud Pública de Oaxaca, Oaxaca 71257, Mexico; 4 Department of Veterinary Integrative Biosciences, Texas A&M University, College Station, TX 77843, USA; 5 Laboratorio de Genética y Genómica Comparativa, Universidad Autónoma de Tamaulipas, Unidad Académica Multidisciplinaria Reynosa-Aztlán, Reynosa 88778, Mexico; 6 Departamento de Microbiología e Inmunología, Facultad de Ciencias Biológicas (FCB), Universidad Autónoma de Nuevo León (UANL), San Nicolás de los Garza, N.L., Monterrey 66455, Mexico

**Keywords:** DNA barcoding, *Trypanosoma cruzi*, domestic triatomines, Oaxaca

## Abstract

**Simple Summary:**

Here, we report entomological and parasitological data on triatomine vectors of *Trypanosoma cruzi*, the agent of Chagas disease, in a highly endemic region of México. The morphological and molecular identification of four principal domestic species, *Triatoma phyllosoma, T. barberi, T. mazzotti,* and *T. dimidiata,* was conducted, and their presence in Oaxaca was documented based on observations from this study in comparison to municipality records from prior published studies. We document the highly endophilic behavior of four triatomine species infected with *T. cruzi*. The present work carried out in Oaxaca was necessary for ongoing efforts to reach the goal of reducing the Chagas disease burden in Mexico and beyond.

**Abstract:**

In Latin America, Mexico is the country with the second highest annual estimated number of Chagas disease cases, caused by *Trypanosoma cruzi*, due to vector-borne transmission. The state of Oaxaca is the location of the first documented human cases of Chagas disease in Mexico and contained the highest *T. cruzi* seropositive rate (3.5%) from blood donors. Here, entomological surveys, from 2017 to 2019, were conducted to collect triatomines in 124 villages of 60 municipalities. Four principal domestic *Triatoma* spp. (Hemiptera: Triatominae), *Triatoma phyllosoma, T. barberi, T. mazzotti,* and *T. dimidiata*, of Oaxaca, Mexico were identified by morphology and molecular analysis of the barcode region of the cytochrome oxidase 1 (cox1 or COI or CO1) gene. A total of 41 out of 83 *T. phyllosoma* specimens examined by microscopy were positive for *T. cruzi* (49%), 49 out of 171 for *T. barberi* (28%), 31 out of 177 for *T. mazzotti* (17%), and none out of 10 for *T. dimidiata* (0%). Overall, the infestation index was 3.1% of households containing at least one triatomine; the crowding index was a mean of two *Triatoma* spp./household; and the colonization index was 0.38 for households based on presence of nymphs. Geographical distribution of triatomines in Oaxaca at the municipality level and endophilic behavior is also reported. Precise identification, endophilic habits, and infection rates of these triatomines are paramount for vector control programs of the Ministry of Health of Oaxaca and beyond.

## 1. Introduction

The World Health Organization [1] reports that over six million people are infected by *Trypanosoma cruzi*, the parasite that causes Chagas disease, for which there are still no vaccines. This protozoan blood parasite, *Trypanosoma cruzi*, is considered a generalist and infects multiple species of wild and domestic mammals [2,3,4]. Reptiles, amphibians, and birds cannot be infected, and are considered refractory due to antigenic incompatibility, but they are food sources for triatomines [2,5]. *Trypanosoma cruzi* can be transmitted orally, congenitally, and through blood transfusions, but vector-borne transmission is the primary mode [6]. Therefore, the correct identification of triatomine vectors and the detection of *T. cruzi* are important steps for the effective control of vector-borne transmission.

In Mexico, Chagas disease is classified into acute and chronic phases [7]. In the Americas, the environmental context where triatomines exist and transmit *T. cruzi* are sylvatic, peridomestic, and domestic cycles [8]. Mexico contains all three contexts and has minimal vector control activities [9]. Mexico ranks third of all Latin American countries for the total estimated number of *T. cruzi*-infected people. In addition, Mexico is second from the top for the estimated annual number of new cases due to vector-borne transmission [10]. From 2001 to 2021, 11,673 cases (acute, indeterminate, and chronic Chagas disease) were registered by the Ministry of Health in Mexico [7]. Veracruz is the state with the highest number of Chagas cases, followed by Jalisco, State of Mexico, Oaxaca, Yucatan, Hidalgo, Chiapas, and Tamaulipas [11]. Oaxaca reported 8.4% (544 cases) of the total cases in Mexico. However, Oaxaca had the highest *T. cruzi* seroprevalence rate of 3.5% from blood donors; therefore, it was estimated that diagnostic tests and treatments for Chagas disease would cost over USD 1.6 million, and it has a burden of disability-adjusted life years of USD 30 million [12]. In the late 1930s in Oaxaca, Dr. Mazzotti discovered the first human cases of Chagas disease of Mexico [13]. 

In Mexico, 32 species and 13 genera of triatomines have been documented, all of which are potentially involved in transmission to humans [14]. Of these, two are reported to colonize intradomicile habitats, *Triatoma dimidiata* and *T. barberi*, and eleven are only known to occur in peridomicile habitats [3,15]. Six triatomine species, *T. barberi, T. dimidiata, T. mazzotti, T. phyllosoma, T. picturata,* and *Rhodnius prolixus*, had been previously reported for Oaxaca [16], with four additional species: one, *T. gerstaeckeri*, found during the 1980 entomological survey [17], and *T. pallidipennis, T. bolivari,* and *T. nitida* found during 1996–1998 surveys [12]. Here, we update the geographical distribution of the four principal and most predominant domestic triatomines of Oaxaca, Mexico, using morphological and molecular identification. We also report the percentage of triatomines harboring *T. cruzi*; household colonization, crowding, and infestation indices; and the vector endophilic behavior in Oaxaca.

## 2. Materials and Methods

### 2.1. Study Area

**Figure 1:**
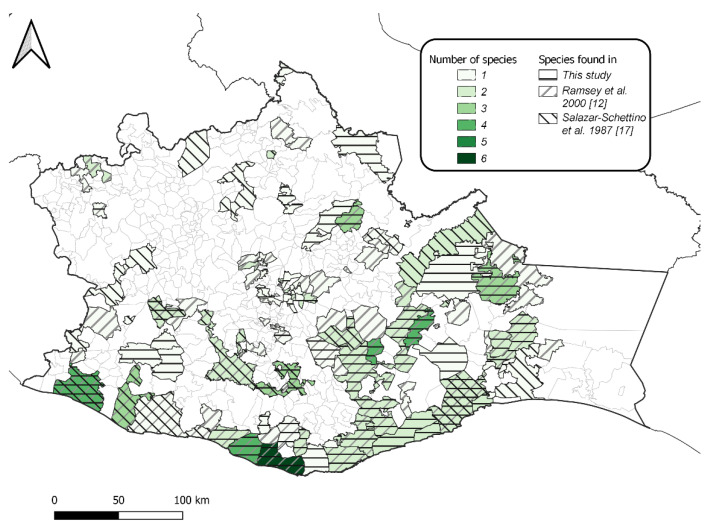
Distribution of the four predominant domestic species of Triatominae in the state of Oaxaca, México: *Triatoma barberi, T. dimidiata, T. mazzotti,* and *T. phyllosoma*; these four, plus *T. phyllosoma picturata, Rh. prolixus*, *T. bolivari, T. gerstaeckeri*, *T. pallidipennis*, and *T. nitida*, were reported in municipalities in Oaxaca by Salazar-Schettino et al. (1987) [17] and Ramsey et al. (2000) [12].

This study was carried out from 2017 to 2019 in Oaxaca (Figure 1), whose geography and demography were already depicted elsewhere [12]. Triatomines were searched for in 124 villages distributed in 60 (10.5%) of the 570 municipalities in Oaxaca. A total of 4000 households/year were examined for triatomines. In each village, a minimum of 8–10 households were sampled. If the village had less than 500 inhabitants, all households were sampled. When the village had over 500 inhabitants and 1000 households, 30% and 10% of households were sampled, respectively. The sampling effort consisted of two persons per household working for 1 h of standard manually timed search for triatomines in the domicile and peridomicile habitats (30 min each), while searching for any stage of triatomines (adults, nymphs, and eggs), dry feces, or exuviae. The collectors also obtained triatomines collected by the household’s owner. The two-person team looked for triatomines in possible shelters inside and outside the house, such as cracks in the walls, ceilings, behind pictures, furniture, calendars and pictures, in the beds, in the door frames, cardboard boxes, materials of construction, firewood and stacked stone, animal pens, and chicken coops. An amount of 12.5 g/L of water of Ficam (Bendiocarb), sprayed with a Hudson pump, served as an irritant that attempted to make the triatomine crawl out of its hiding location. Entomological forceps, never the bare hands, were used to collect the triatomines [18]. Specimens were kept alive (two per household) in wide-mouth jars containing folded cardboard and labeled with field data. One bottle was used for triatomines found in the peridomicile, and another was used for those found inside the household (intradomicile). The two bottles were transported to the nearest sanitary jurisdiction, and later, to the Oaxaca state lab, where fecal collection and the search for the parasite were carried out promptly, i.e., within three days maximum post-collection of the triatomines, which were kept alive under standard insectary conditions. The first instar nymphs were not considered in the analysis and reporting, as they likely hatched during their transport from field to lab. In addition, first instar nymphs start feeding after 3–4 days post-hatching, so it is unlikely that they harbor *T. cruzi*. 

### 2.2. Morphological Identification and Molecular Analysis

The identification of triatomines was performed by morphological keys [19]. For the molecular characterization of triatomines, genomic DNA extraction was carried out using a slightly modified non-destructive extraction method, as described by Truett et al. (2000) [20]. Briefly, tissue of individual triatomines and/or their genitalia was placed into individual 200 μL PCR tubes containing 20 μL of alkaline lysis buffer (25 mM NaOH and 0.2 mM EDTA), incubated at 94 °C for 30 min, and then at 4 °C for 5 min to cool down. After vortexing, 20 μL of neutralizing buffer (40 mM Tris-HCl) was added. After mixing, the tubes were centrifuged at 12,000 rpm for 15 min, and then the supernatant was transferred to a new tube for PCR for the barcode region of the cytochrome oxidase 1 (cox1 or COI or CO1), whose amplicon size was 658 bp. The PCR conditions and protocol used were as reported elsewhere [21]. PCR products were separated by 1% agarose gel electrophoresis and purified using the QIAquick PCR Purification Kit (QIAGEN, Montgomery County, MA, USA). PCR products were sent out to Eurofins, purified again, and sequenced in both directions by Eurofins (Eurofins, Genomics, Louisville, KY, USA). We trimmed off the ends that had low-quality sequences based on chromatographs. The sequences were manually edited using BioEdit v. 7.0.5.3 [22]. The species were identified, using the Basic Local Alignment Search Tool (Available online: https://blast.ncbi.nlm.nih.gov, accessed on 29 November 2022) [23,24], according to the percentage of nucleotide identity. In addition, sequences and sampling data of triatomines were uploaded to the Barcode of Life Data System [25,26] under the project entitled: Triatominae of Mexico (code = TRTMX; accession numbers 19001-19110).

### 2.3. Parasitological Examination

The abdominal compression of live insects was applied to collect feces on glass slides [27]. The feces were diluted with 50 µL of PBS solution, pH 7.0, and parasites were observed at 40× through an optical microscope (Olympus CH2, Tokyo, Japan) [28,29]. As shown in Table 1, 31.9% of the triatomines processed did not yield feces, so the parasite detection was not able to be performed.

### 2.4. Geographical Distribution

Distribution maps of triatomines were created using geographic information systems (QGIS) v. 3.22.13 LTR (Available online: https://qgis.org/en/site/, accessed on 29 November 2022) and vector layers in the shape format of the Basic Geostatistical Framework 2020 from National Institute of Statistics, Geography and Informatics (INEGI), México.

### 2.5. Statistical Analysis

The 95% exact Bayes confidence intervals (95% CIs) surrounding the point estimate of vector infection prevalence was calculated as previously reported [30]. Entomological indices were also calculated [18]. The infestation index = No. of households containing triatomines/total No. of households × 100; the colonization index = No. of households harboring nymphs/total No. of households with triatomines × 100; and the crowding index = total No. of triatomines/total No. of households with triatomines.

## 3. Results

### 3.1. Morphological and Molecular Identification

Triatomines were searched for in 124 villages of 60 municipalities in Oaxaca (Figure 1) from 2017–2019. A total of 4000 households/year were examined, where 381 households were found to contain triatomines (overall infestation index = 3.1%; i.e., the percentage of households with triatomines). This study reports 32 new municipality records for the presence of triatomines in Oaxaca (Figure 1). A total of 774 triatomines were collected, representing nymphal instars from I to IV (first instar nymphs were not considered in the analysis and reporting) and both adult females and males (Table 1) from 381 households sampled containing at least one triatomine. After morphological identification was performed, 116 individuals were randomly selected from the different morpho-types for barcoding. Of these, 85 individuals, consisting of 13, 1, 39, and 32 individuals of *T. barberi*, *T. dimidiata*, *T. mazzotti,* and *T. phyllosoma*, respectively, were subjected to DNA extractions. PCR amplicons for a CO1 fragment were produced; however, as the PCR amplicons were purified twice, the DNA concentration was reduced for successful sequencing. Thus, 26 of the specimens examined had a CO1 sequence from which the identities of two species, *T. mazzotti* and *T. phyllosoma*, were confirmed in BOLD [26] or by NCBI-BLAST. Most barcode sequences longer than 500 bp were assigned a Barcode Index Number (BIN), a taxonomic system that assigns similar barcode sequences into species proxies without the need for Linnaean nomenclature [31]. Thus, from 13 sequences (with a range of 505–645 bp) of *T. mazzotti* uploaded to BOLD, five BINs, AEG2827, AEG3814, AEG3815, AEG9326, and ABY0975, were assigned by the algorithm of BOLD. From seven sequences of *T. phyllosoma* uploaded to BOLD (with a range of 512–620 bp), four BINs, AEG8834, AAJ8726, AEG3041, and AAJ8726, were also assigned. The remaining six sequences of morpho-types *T. mazzotti* (two individuals) and *T. phyllosoma* (four individuals) were identified, using NCBI-BLAST, as *T. phyllosoma* (percent identity range = 95.12–98.98%; accession number DQ198806.1).

Although one sequence of *T. dimidiata* was uploaded to BOLD, no BIN was assigned, because the sequence was of 312 bp, so the identity of this species could not be confirmed in BOLD. However, using NCBI BLAST (Available online: https://blast.ncbi.nlm.nih.gov, accessed on 29 November 2022), the nucleotide percent identity was 98.0% to a sequence of *T. dimidiata* isolated from Veracruz in 2020 (accession number MT556666.1) [32]. Although 13 adult individuals of *T. barberi* were subjected to DNA extractions, and PCR amplicons were obtained, we did not successfully obtain sequences.

In addition, nine adult individuals not able to be identified morphologically to species level were subjected to molecular barcoding. Two individuals produced a sequence of 578 bp and 645 bp and were assigned the BIN ABY0975 and, therefore, were identified as *T. mazzotti*. Two additional sequences of 522 and 593 bp were assigned the BIN AEG8834 and, therefore, were identified as *T. phyllosoma*. These BINs were assigned algorithmically by BOLD, indicating the utility of the tool for helping in the establishment of species boundaries based on CO1 DNA barcode sequences. One individual was identified by NCBI-BLAST as *T. phyllosoma* (percent identity 96.3%; accession number = DQ198806.1). Although PCR amplicons were obtained from the remaining four adult individuals, no sequence was produced, because a suboptimal DNA concentration was used for sequencing.

Here, we also tested DNA sequencing for its ability to identify nymphs of unidentified triatomine species of Oaxaca. A random subset of 22 unidentified *Triatoma* spp. nymphs was also subjected to DNA extractions, and PCR amplicons were obtained; however, as aforementioned, the DNA concentration was not optimal for successful sequencing for the 22 samples. Thus, only four samples produced a PCR amplicon for a CO1 fragment and were sequenced. One had a CO1 sequence of 545 bp; thus, it was identified by molecular analysis as *T. mazzotti* (BIN = AEG3815). Another two sequences of 358 and 391 bp were not assigned a BIN in BOLD; however, using NCBI-BLAST, the nucleotide percent identity was 99.1% and 97.9%, respectively, to sequences of *T. phyllosoma* EP-05-007 CIAD (accession number DQ198806.1 [33]). The other one was a failed sequence. On the whole, 116 individuals were subjected to DNA extractions, and 92 produced PCR amplicons (i.e., with a PCR success rate of 79.3%). Out of this sample, only 36 sequences were produced as aforementioned (i.e., with a sequencing success rate of 39%).

### 3.2. Endophilic Behavior

On the whole, 32 out of the 60 municipalities of Oaxaca searched in this study represent new municipality records for the presence of triatomines (Figure 1). Overall, one-hundred and three triatomines were found in bedrooms of the 381 positive households sampled (27%; 95% CIs =22–31%). *Triatoma mazzotti* and *T. phyllosoma* have been previously documented to have peridomicile habits; however, in 74 out of 167 (44%) households where *T. mazzotti* was found, it was localized in the bedroom. In 5 out of 57 (9%) households where *T. phyllosoma* was found, it was in the bedroom. In addition, 93 triatomines of the 4 primary domestic species were also found inside the households (Table 2), but in other sites of the household, such as on the wall, floor, sofa, in the kitchen, living room, bathroom, in box containing books, or in dirty clothes, indicating synanthropism and highly endophilic behavior. However, 176 triatomines were also found around the peridomicile on firewood, wood, brick, patio, stone, corral, in the hen house, and in rubbish.

### 3.3. Entomological Indices

Overall, the colonization index was 0.39 (range = 0.03–0.72; 151 households contained nymphs of *Triatoma* spp./381 positive households), the crowding index was a mean of 2.0 (774/381) *Triatoma* spp. per household (range = 1.1–4.3; Table 1), and the infestation index = 3.1% of households with *Triatoma* spp. from the total sample. *Triatoma barberi* had the highest colonization and crowding indices of 0.72 and 4.3, respectively, but its infectivity rate of 28% (95% CIs = 22–35%) was lower (*p* < 0.05) than that of *T. phyllosoma*, which was 49% (95% CIs = 38–60%), with entomological indices of 0.14 and 2, respectively. *Triatoma mazzotti* less frequently colonized (index = 0.03) human dwellings (with a crowding index of 1.1), but its infectivity rate of 17% (95% CIs = 12–23%) was similar (*p* < 0.05) to that of *T. barberi*.

Overall, the infection rate of 43.5% (with 95% CIs of 38.5–48.6%) in the peridomicile triatomines was similar (*p* < 0.05) to that of the intradomicile triatomines, which was 33.6% (with 95% CIs of 26–41.4%), as the CIs did overlap. In addition, 333 unidentified nymphs of *Triatoma* spp. specimens were examined, and 98 were positive for *T. cruzi,* representing an infectivity rate of 29% (with 95% CIs of 24–34%; Table 1). The only nymph of *T. mazzotti* identified by molecular analysis in BOLD in this study was negative to *T. cruzi* and was collected in a peridomicile environment. One nymph of *T. phyllosoma*, using NCBI-BLAST, was positive for *T. cruzi* and was collected from the intradomicile environment.

## 4. Discussion

**Figure 2:**
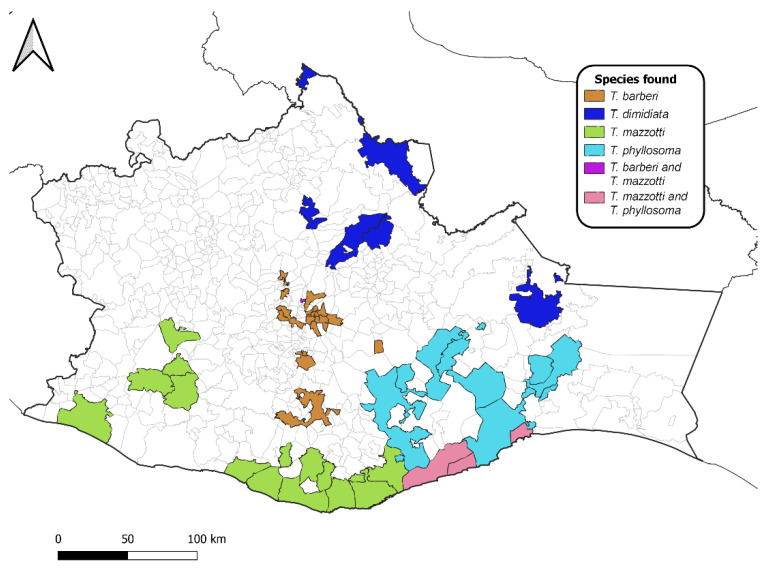
Actual distribution of the four predominant domestic species of Triatominae in the state of Oaxaca, México: *Triatoma barberi*, *T. dimidiata*, *T. mazzotti*, and *T. phyllosoma* (including *T. phyllosoma picturata*).

This study demonstrated that Chagas disease is still of paramount importance in Oaxaca. In our entomological surveys, we found that *Triatoma* spp. nymphs, which are difficult to identify morphologically, could be identified by DNA barcoding, similar to what has been utilized for the molecular identification of immature *Culicoides* biting midges [34]. Past entomological surveys of the four primary domestic triatomines of this region, *T. dimidiata, T. mazzotti, T. pallidipennis,* and *T. phyllosoma*, in 1980 documented their occurrence in 24 municipalities [17]; however, Ramsey et al. (2000) [12] conducted a study between 1996–1998 and reported eight species of triatomines in 64 municipalities [12]. The current study also documents records of the four primary domestic triatomines in 60 municipalities (Figure 1). Figure 1 depicts exactly the 32 new municipality records of this study, revealing, in terms of vector, species occurrence, while Figure 2 shows the actual presence of *Triatoma barberi, T. dimidiata, T. mazzotti,* and *T. phyllosoma*. We also highlighted the historic distribution records of the four predominant domestic species of triatomine (Figure S1–S4) and of other triatomine species (Figure S5) [12,17].

As the four primary domestic triatomines of this study were predominantly found in bedrooms, these data may suggest an adaptation to households for four triatomine species, only two of which, *T. dimidiata* and *T. barberi*, were previously documented in domestic settings [17]. Our estimated colonization index of 0.39 indicates persistent infestation of new generations of *Triatomas* spp. within households of Oaxaca. Our results were similar to the overall colonization index of 0.55 (with a range of 0.3–0.9) reported by Ramsey et al. (2000) in this same region, 19 years before our study [12]. This evidence of persistent domiciliary colonization highlights the necessity for intensified vector control. The crowding and infestation indices of 2.0 *Triatoma* spp. per household and 3.1% of households with *Triatoma* spp. (<8% is considered a low infestation rate [35]), respectively, were lower than the overall values of 5.9 and 7% (range = 5.1–8.2%)*,* respectively, for *T. phyllosoma*, reported by Ramsey et al. (2000) [12]. However, vector index thresholds for control may differ between epidemiological settings.

In Mexico, Vidal-Acosta et al. (2000) [5] examined 5399 specimens, of which 13 species were associated with households; 8 species were found infected: *T. mexicana, T. dimidiata, T. barberi, T. mazzotti, T. pallidipennis, T. picturata, Rhodnius prolixus,* and *T. longipennis.* The vectors in the states of Morelos, Michoacán, and Nayarit, Mexico had the highest infection rates (50%, 36.4%, and 29.2%, respectively), with new state and local records of *T. dimidiata, T. gerstaeckeri, T. longipennis, T. mexicana, T. pallidipennis*, and *Pastrongylus rufotuberculatus* [5]. In Chiapas, *T. cruzi*-infected *T. dimidiata* were documented for the first time inside an urban dwelling in Tuxtla Gutiérrez by De Fuentes-Vicente (2021) et al. [36], indicating that this species in southern Mexico may be spreading into urban areas, similar to what has been observed in Perú by Delgado et al. (2013) [37]. In Guerrero, 71 (32.4%) of collected triatomines were found infected with *T. cruzi*, but only *T. pallidipennis* was reported by Becerril-Flores (2003) [38]. Twenty-seven (53%) municipalities of Jalisco had infected triatomines out of eight species collected by Magallón-Gastelum et al. (1998) [39]. In Yucatán, an overall *T. cruzi* infection rate of 34% was reported for *T. barberi* by Dumonteil et al. (2002) [40]. These results were in concordance with our study, as the infectivity rate of *T. barberi* was 28% (with 95% CIs of 22–35%; Table 1).

Rojas et al. (1989) [41], in ‘La Humedad’, Santiago Jamiltepec, Oaxaca, found *T. mazzotti* infected with *T. cruzi* in households, and in four small caves with bats and other mammals. The *T. cruzi* parasite infectivity rate of *T. dimidiata* was reported to be 3.7% in Oaxaca by Ramsey et al. (2000) [12], but in our study, we did not find any *T. dimidiata* infected with the parasite. Ramsey et al. (2000) [12] reported a *T. cruzi* infectivity rate for *T. phyllosoma* of 27.2%, which is lower than that of the 49% found in the current study.

A limitation of the current study is that we were unable to confirm the sensitivity of parasite detection performed by microscopy versus molecular testing, and the true infection prevalence of the four principal domestic triatomines of Oaxaca may be greater than what we report. Perez-España et al. (2019) [28] estimated an overall infectivity rate of 1.76% from 170 *Triatoma* spp. (*T. dimidiata, T. mexicana,* and *T. gerstaeckeri*) when examined using traditional microscopy, but the rate was 11.17% using PCR. Additionally, although the identification of *T. cruzi* was conducted by co-author (J.C.T.G.), who is highly trained and certified by Instituto de Diagnóstico y Referencia Epidemiológicos (InDRE), Ministry of Health, the presence of other trypanosomes, such as *T. rangeli* or *Blastocrithidia* spp., cannot be ruled out [42]. Salivary glands of triatomines collected in Oaxaca were examined for possible *T. rangeli* infection by Ramsey et al. (2000) [12], but no further *T. rangeli* evidence of this parasite in those vectors in Oaxaca was reported by Ramsey et al. (2000) [12].

Technical failures of the DNA processing protocol, which resulted in a low success rate of insect barcoding, was also a limitation. DNA extraction methods are available to recover longer DNA fragments from degraded samples, such as commercial kits (DNeasy Blood & Tissue Kit from QIAGEN, Montgomery County, Maryland, United States) and Chelex and cetyltrimethylammonium bromide (CTAB); this study aimed to use a non-destructive, inexpensive, modified Hotshot technique that has proved to be useful for the barcoding of mosquitoes and sandflies [43,44]. Tissue (legs) of individual triatomines 1–2 years old, stored dry at room temperature, was used first, but no genomic DNA was recovered. However, when the genitalia were used, this produced genomic DNA, with a PCR success rate of 79.3%. An earlier experiment, using 13 older specimens of triatomines collected in the same villages of Oaxaca, produced a sequencing success rate of 100%, with barcodes (407–658 bp in length) recovered from five morphospecies (unpublished data); however, DNA extractions were automated using a 96-multichannel Biomek NX robotic liquid handler (Beckman Coulter Inc., Brea, California, United States) with a Thermo Cytomat hotel and PCR, and sequencing was performed in-house at the Centre for Biodiversity Genomics of the University of Guelph, Canada. The low sequencing success rate of 39% of this study was likely due to the double purification step of the PCR amplicons.

Currently, national elimination programs for Chagas disease in Mexico are underway [7] and have been operating for over 20 years. Triatomine populations are known to change over time and colonize new habitats, which emphasizes the importance of the current study for updating Oaxaca’s *Triatoma* spp. infectivity rate with *T. cruzi* and household infestation, crowding, and colonization indices. Oaxaca continues to be one of the leading states in Mexico for the number of human cases of Chagas disease. Oaxaca also has a high diversity of vector species, with both domicile and peridomicile habits, as shown here, which persist despite vector control activities using insecticides. This context warrants insecticide resistance monitoring to ensure the products being used remain effective, but also the consideration of alternative vector control tools, such as bed nets and household improvements [45,46]. A program for combating Chagas disease in Oaxaca, which includes searching for cases in children 15 years old and under, which are assumed to be locally acquired, is currently underway to discover hotspots. Improved surveillance will help monitor control activities and direct resources to areas of greatest need in Oaxaca and globally.

## Figures and Tables

**Table 1:** Entomological and parasitological indices for the four predominant domestic triatomines of Oaxaca, México.

Species	No. of Municipalities	No. of Positive Households Sampled	Colonization Index ^&^	Crowding Index ^$^	No. of Infected Triatomines/No. Examined	Infectivity Rate CI *
*T. phyllosoma*	12	57	0.14	2.0	41/83	49% (38–60%)
*T. barberi*	20	77	0.72	4.3	49/171	28% (22–35%)
*T. mazzotti*	21	167	0.03	1.1	31/177	17% (12–23%)
*T. dimidiata*	7	8	0.50	1.4	0/10	0% (17% ^#^)
Unidentified *Triatoma* spp.	25	72	1.0	1.8	98/333	29% (24–34%)
Overall	60	381	0.38	2.0	219/774	28% (25–31%)

^&^ No. of households that contained nymphs of *Triatoma* spp./number of positive households. ^$^ Mean number of *Triatoma* sp. per household. * Point estimates and 95% confidence intervals (CI) are shown. ^#^ As point estimate was 0, only the upper limit of CI was calculated.

**Table 2:** *Triatoma* spp. infected or not with *Trypanosoma cruzi* found in intradomicile or peridomicile in Oaxaca, México.

			Adult Life Stage								
No. of Households Sampled	Collection Site		*T. mazzotti*	*T. barberi*	*T. phyllosoma*	*T. dimidiata*	Unidentified *Triatoma* spp.				Total
							Nymph Stage *				
							V	IV	III	II	
196	Intradomicile	+	18	2	16	0	6	3	2	1	48+
		−	45	1	14	1	11	12	11	0	95−
		NF	69	3	9	5	5	8	8	4	111 NF
176	Peridomicile	+	13	47	25	0	14	28	26	6	159+
		−	7	65	10	1	29	31	48	15	206−
		NF	20	53	7	2	14	10	17	4	127 NF
9	ND	+	0	0	0	0	2	4	2	4	12+
		−	0	0	2	0	1	1	0	3	7−
		NF	5	0	0	1	0	1	1	1	9 NF
381	Total		177	171	83	10	82	98	115	38	774

+ = individuals harboring *T. cruzi*; − = individuals free of infection; NF = no feces; ND = no data. * only nymph stages II-IV were examined; we did not include stage I nymphs in the *T. cruzi* testing, since they are unlikely to be positive, and it would be harder to obtain feces from them.

## Data Availability

Public data of triatomines can be found in the Barcode of Life Data System using the word Triatoma and Taxonomy in the search tool (Available on: https://www.boldsystems.org/index.php/Taxbrowser_Taxonpage?searchMenu=taxonomy&query=triatoma&taxon=triatoma, accessed on 29 November 2022). Public BINs of Triatoma of Oaxaca (AAJ8726, ABY0975, and AEG3815) can be found in the Barcode of Life Data System using BINs and TRTMX in the search tool (Available on: https://www.boldsystems.org/index.php/Public_BINSearch?searchMenu=bins&query=TRTMX&taxon=; accessed on 29 November 2022).

## Supplementary Materials

The following supporting information can be downloaded at: https://www.mdpi.com/article/10.3390/insects13121134/s1, Figures S1–S5. Historic distribution of the four predominant domestic species of Triatomine (Figures S1–S4) in the state of Oaxaca, México: Figure S1. Historic distribution of *Triatoma barberi*; Figure S2. Historic distribution of *T. dimidiata*; Figure S3. Historic distribution of *T. mazzotti*; Figure S4. Historic distribution of *T. phyllosoma* (including *T. phyllosoma picturata*); and other five species; Figure S5. Historic distribution of *T. gestaeckeri*, *T. bolivari*, *T. pallidipennis*, *T. nitida*, and *Rhodnius prolixus* from previous studies [12,17].
