# Supplementary figures and images for: Domestic Triatoma spp. Infections with Trypanosoma cruzi, Household Infestations, and Molecular Identification in Oaxaca, México
Source: Insects. 2022 Dec 8;13(12):1134. doi: 10.3390/insects13121134 (PMC9785609; doi:10.3390/insects13121134)

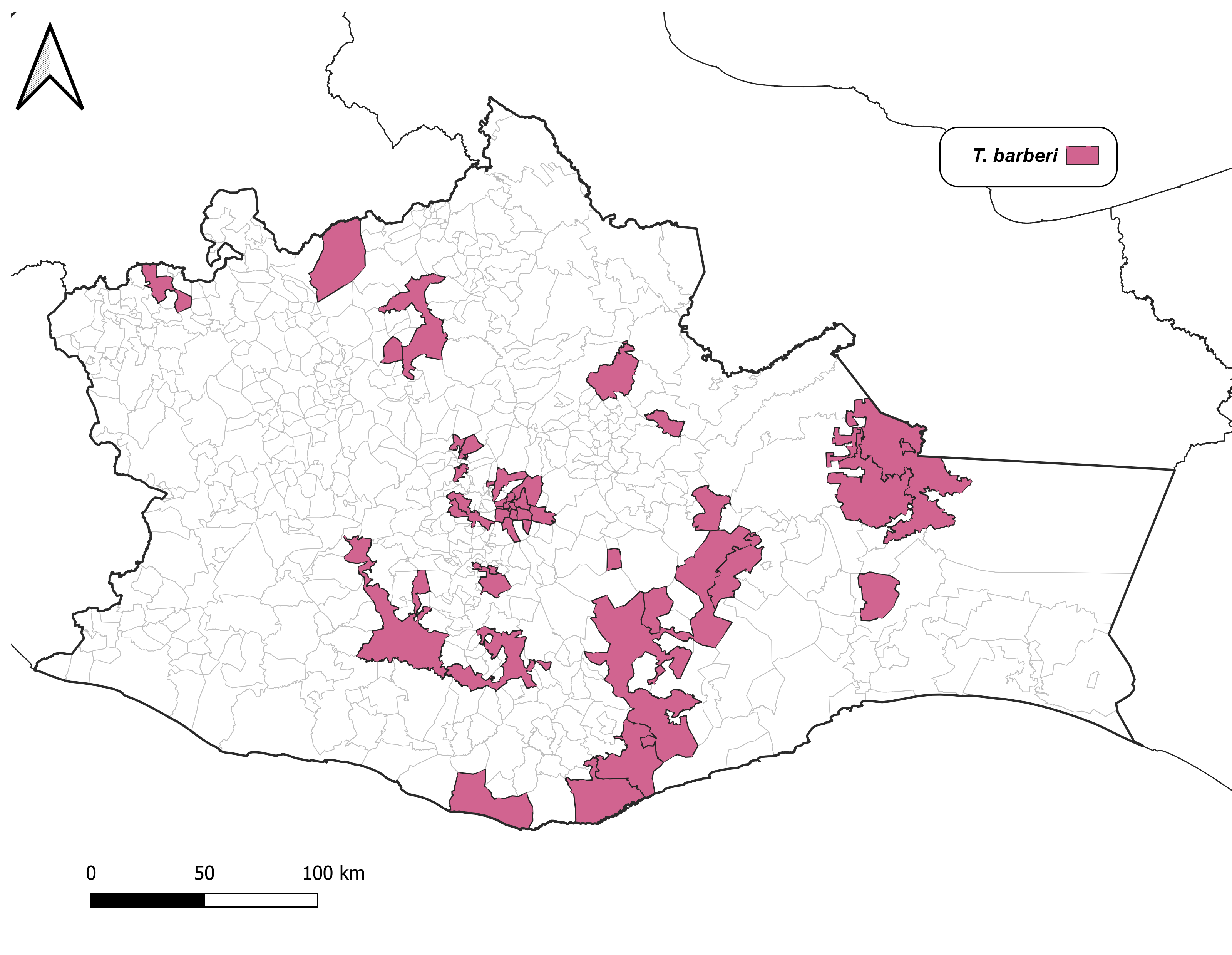

Supplement: Supplementary file 1 [file insects-13-01134-s001.zip › insects-2075511-supplementary/Figure S1.png]

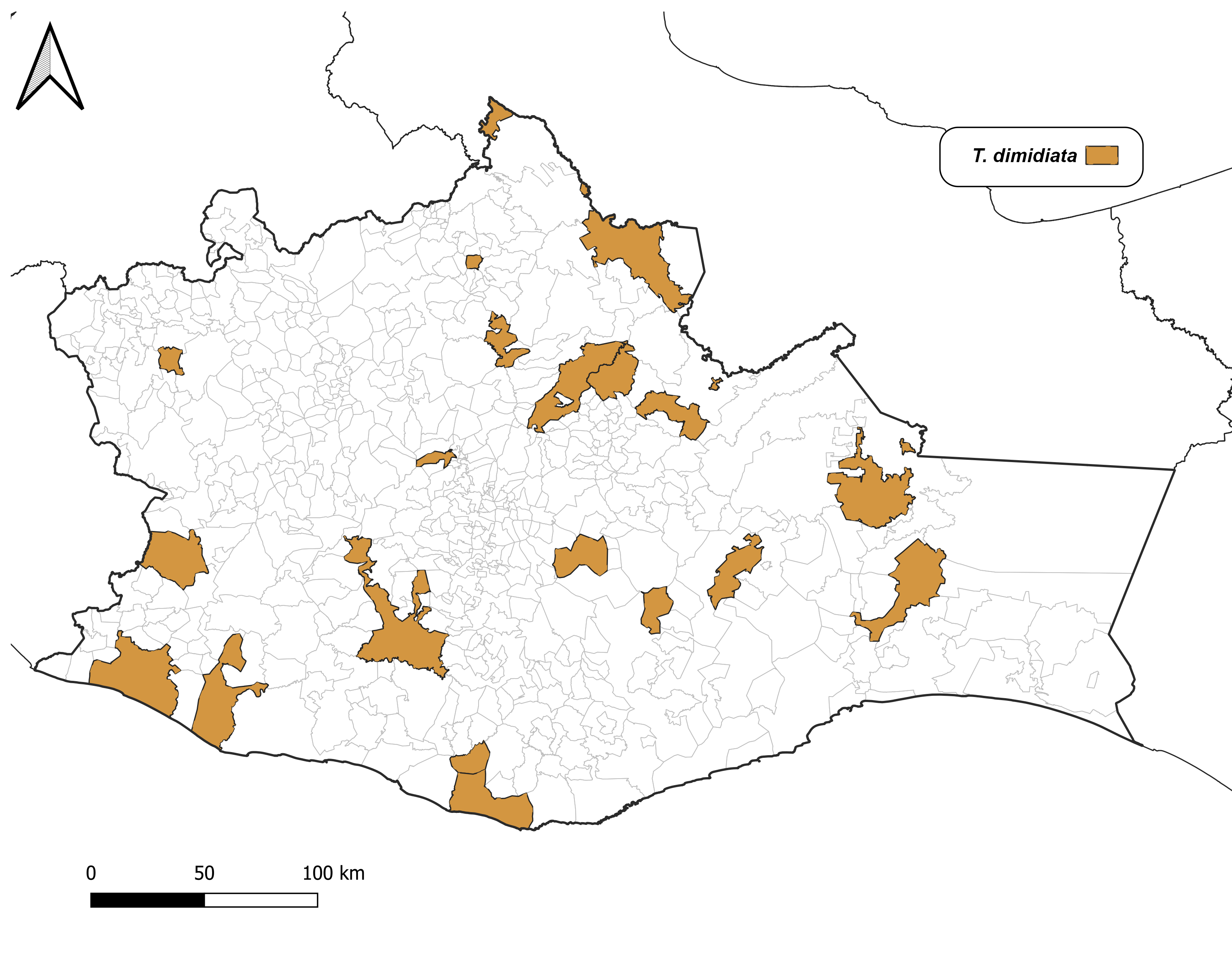

Supplement: Supplementary file 1 [file insects-13-01134-s001.zip › insects-2075511-supplementary/Figure S2.png]

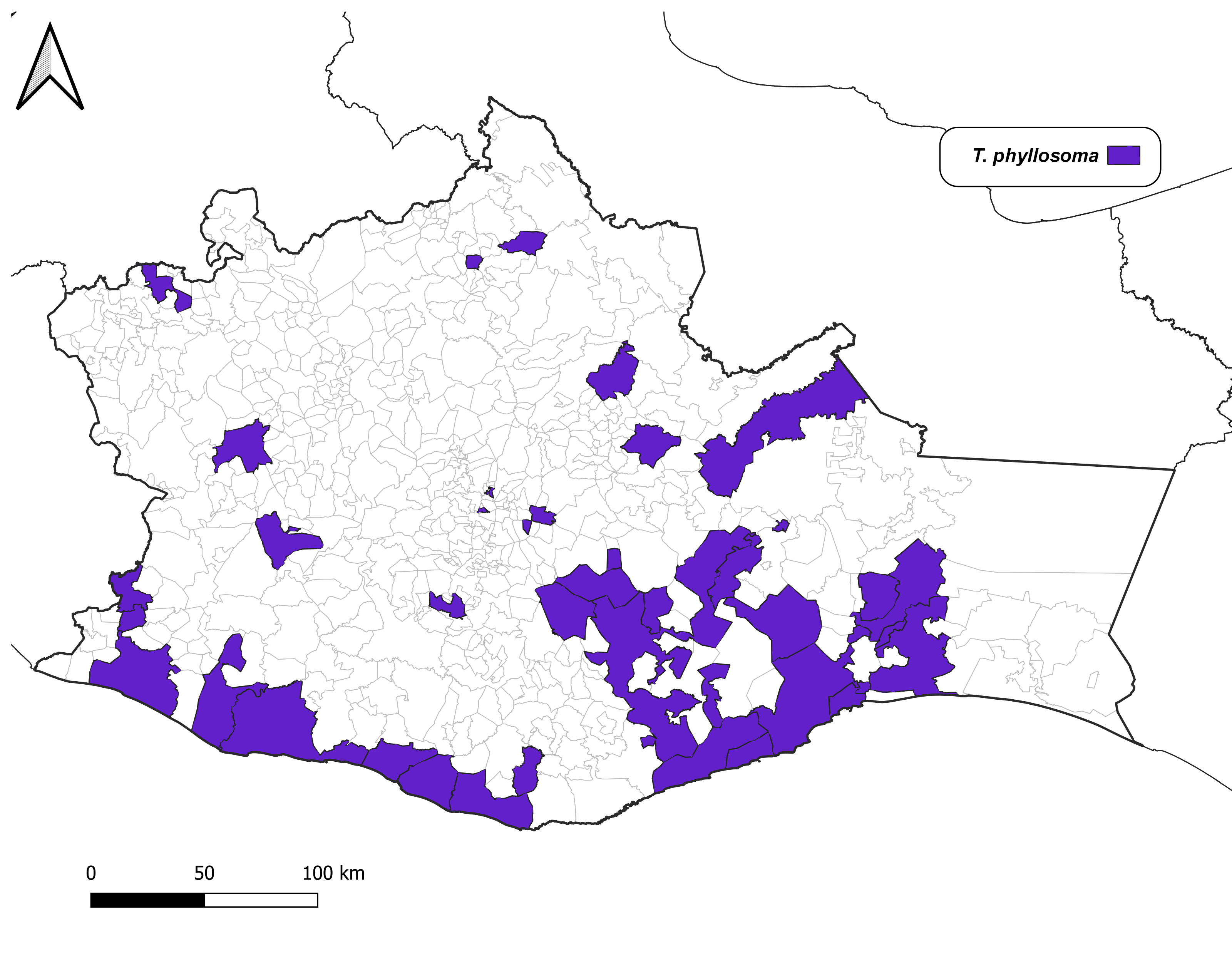

Supplement: Supplementary file 1 [file insects-13-01134-s001.zip › insects-2075511-supplementary/Figure S3.png]

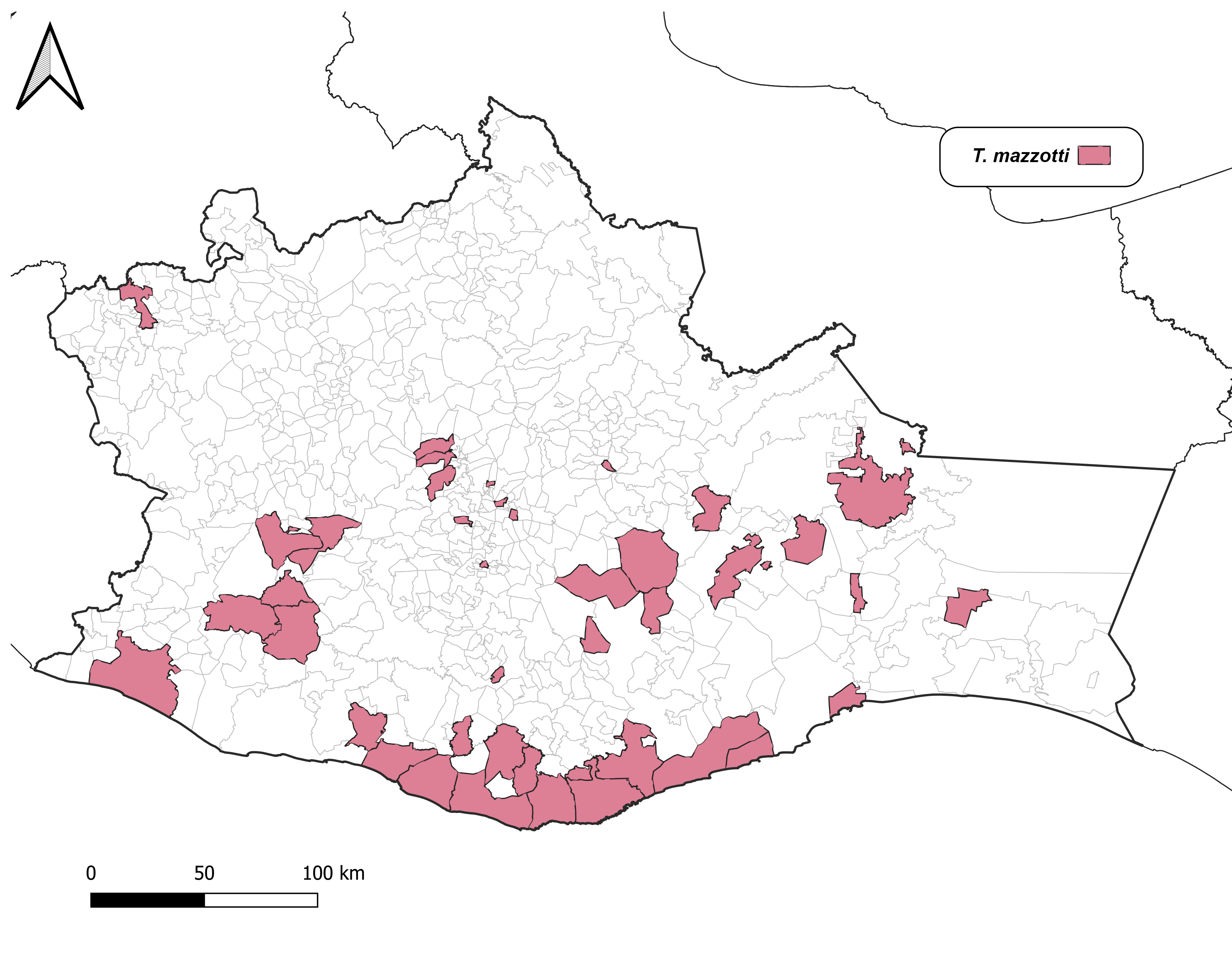

Supplement: Supplementary file 1 [file insects-13-01134-s001.zip › insects-2075511-supplementary/Figure S4.png]

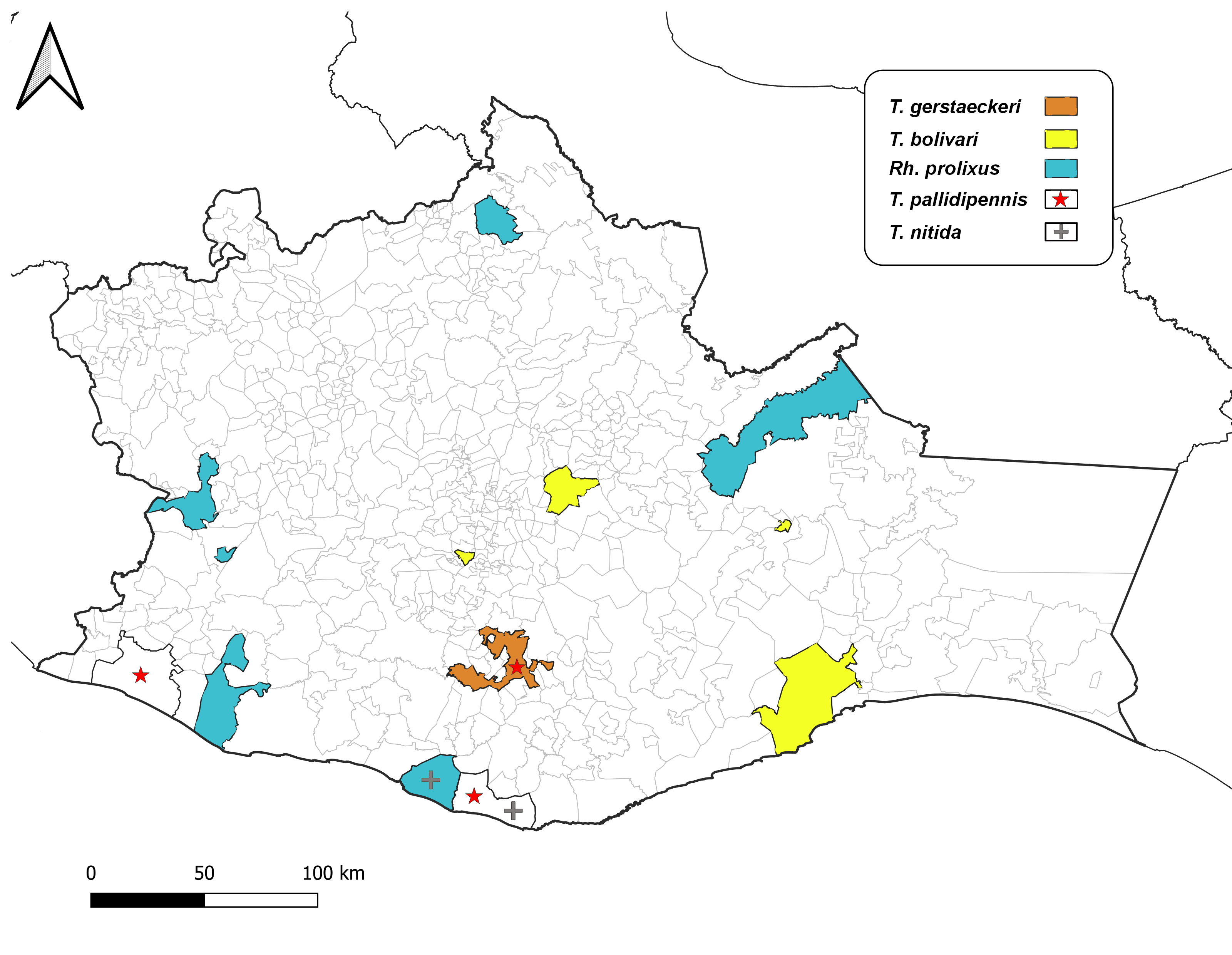

Supplement: Supplementary file 1 [file insects-13-01134-s001.zip › insects-2075511-supplementary/Figure S5.png]
